# Effects of Long-Term Antiglaucoma Eye Drops on Conjunctival Structures: An In Vivo Confocal Microscopy Study

**DOI:** 10.1155/2015/165475

**Published:** 2015-06-15

**Authors:** Wenqing Zhu, Xiangmei Kong, Jianjiang Xu, Xinghuai Sun

**Affiliations:** ^1^Department of Ophthalmology & Visual Science, Eye & ENT Hospital, Shanghai Medical College, Fudan University, Shanghai 200031, China; ^2^State Key Laboratory of Medical Neurobiology, Institutes of Brain Science, Fudan University, Shanghai 200032, China; ^3^Key Laboratory of Myopia, Ministry of Health, Fudan University, Shanghai 200031, China; ^4^Shanghai Key Laboratory of Visual Impairment and Restoration, Fudan University, Shanghai 200032, China

## Abstract

*Purpose*. The study was aimed at comparing the long-term effects of different antiglaucoma eye drops on conjunctival structures using laser scanning confocal microscopy.* Methods*. Eighty patients diagnosed with primary open-angle glaucoma and twenty healthy volunteers were included in this study. The participants were divided into 5 groups according to the different medications. The lachrymal film break-up time, Schirmer's I test, and Ocular Surface Disease Index Questionnaire were performed in all subjects. The confocal microscopy was used to observe the basal epithelial cell density (ECD), goblet cell density (GCD), dendritic cell density (DCD), and subepithelial collagen fiber diameter (SFD).* Results*. Statistically significant differences were found among the control group and the antiglaucoma therapy groups in the values of three clinical data (*P* < 0.05). The GCD, DCD, and SFD showed significant differences in all glaucoma groups when compared to the control (*P* < 0.001). Moreover, the prostaglandin group differed from the other antiglaucoma therapy groups in the GCD and SFD (*P* < 0.05).* Conclusions*. Our study confirmed the significant differences in the conjunctival structures based on the effects of antiglaucoma medications. Less pronounced changes were found in the patients treated with prostaglandin analogue than in the other kinds of antiglaucoma therapies.

## 1. Introduction

Glaucoma is a chronic, progressive, optic neuropathy requiring the long-term use of antiglaucoma eye drops. These medications containing preservatives may induce ocular damage, such as squamous metaplasia, subconjunctival fibrosis, and a decrease in goblet cells [1]. A correlation has been found between preservatives and dry eye, in two drugs with 39% dry eye and three drugs with 43% dry eye [2, 3]. Additionally, it was reported that the inflammatory reaction of tear modification might be influenced by antiglaucoma medications [4]. However, the long-term use of antiglaucoma eye drops with preservatives is inevitable, since preservative-free antiglaucoma eye drops are not provided in many developing countries. Therefore, it is of utmost importance to compare the effects of various antiglaucoma therapies with preservatives, which likely stimulate inflammation, on the ocular surface. Human leucocyte antigen expression, a marker of inflammation, was confirmed to be slightly higher in the patients treated with preservative-free timolol than in the control [5]. Moreover, preserved latanoprost causes an increase in human leucocyte antigen expression, when compared with preservatives alone [6].

The conjunctiva contributes to the tear mucous layer and regulates the immune system of the ocular surface. The long-term use of antiglaucoma eye drops has a negative influence on conjunctival structures, including the goblet cells, epithelial cells, dendritic cells, and the subepithelial collagen fibers. Ex vivo studies have demonstrated that topical antiglaucoma eye drops induce structural aberrations of the conjunctiva, including a decline in goblet cells, and an increase in inflammatory cells, squamous metaplasia, and subconjunctival fibrosis [7, 8].

In recent years, the application of laser scanning confocal microscopy (LSCM) has provided a promising method to study the structures of the ocular surface in glaucoma [9–12]. Recent studies have demonstrated that, compared with patients using preservative-free eye drops, aberrations were found in the corneal structures and tear function in the patients using preserved medications [13, 14]. Previous studies have utilized the technique of capturing images of the conjunctival structures [15, 16], while others have confirmed the coherence of the goblet cell density (GCD) in different pathological statuses between the LSCM and impression cytology methods [17, 18]. Mastropasqua and colleagues demonstrated that the density of goblet cells was significantly higher in glaucomatous patients treated with preservative-free medication than in those treated with preservative containing medications using the methods of both LSCM and impression cytology [19]. However, there is a deficit in the literature comparing the effects of different kinds of antiglaucoma eye drops with preservatives on conjunctiva in vivo, with regard to the epithelium, the inflammatory cells, and the collagen fibers. Monitoring conjunctival structure may be much more valuable in evaluating the side effects of antiglaucoma medications and providing reliable evidence for the administration of antiglaucoma therapy, especially in developing countries where preservative-free antiglaucoma eye drops are not provided.

This study aimed to evaluate conjunctival structures, including the epithelial cell density (ECD), GCD, dendritic cell density (DCD), and subepithelial collagen fiber diameter (SFD), using LSCM, and to assess the tear function using Schirmer's I test (ST) and the lachrymal film break-up time (BUT) in subjects exhibiting the long-term use of topical antiglaucoma therapy. Furthermore, there has been much interest in comparing the differences in the conjunctival structure and tear function with various topical antiglaucoma medications.

## 2. Materials and Methods

### 2.1. Study Subjects

This cross-sectional observational study was conducted on 80 patients diagnosed with primary open-angle glaucoma (POAG) and 20 healthy age-matched volunteers. The subjects were divided into 5 groups based on the antiglaucoma eye drops that they used as follows.

*Group 1* (normal group) included 20 eyes of 20 healthy volunteers (average age: 64.1 years, ranging from 29 to 81 years; male/female: 12/8) in accordance with the following criteria: no history of ocular trauma or surgery, no current or long-term ocular eye drop use, no allergic mucosal pathology, no contact lens use, and the absence of ocular or systemic diseases that may have affected the conjunctiva.

*Group 2* (beta-blocker group) included 20 eyes of 20 patients (age: 60.0 years, ranging from 30 to 76 years; male/female: 12/8) accepting treatment with carteolol hydrochloride 2% (Mikelan; Otsuka, Tokushima, Japan) twice daily.

*Group 3* (alpha adrenergic agonist group) included 18 eyes of 18 patients (age: 62.6 years, ranging from 38 to 79 years; male/female: 11/7) accepting treatment with brimonidine tartrate 0.2% (Alphagan; Allergan, California, USA) twice daily.

*Group 4* (prostaglandin group) included 21 eyes of 21 patients (age: 61.2 years, ranging from 32 to 80 years; male/female: 14/7) using Travoprost 0.004% (Travoprost; Alcon, Texas, USA) once daily.

*Group 5* (combination therapy group) included 21 eyes of 21 patients (age: 63.5 years, ranging from 23 to 87 years; male/female: 13/8) accepting treatment with two or three antiglaucoma eye drops, including a beta-blocker, alpha adrenergic agonist, and prostaglandin analogue.

Groups 2 through 5 met the following inclusion criteria: the diagnosis of POAG treated with the indicated topical antiglaucoma medications for at least 6 months, without changes. The diseases of the patients were all well controlled with medical therapy, and glaucoma was defined according to the criteria set forth by the International Society for Geographical and Epidemiological Ophthalmology [20]. The exclusion criteria included ocular or systemic diseases that may have affected the conjunctiva, current use of contact lenses, and a history of ocular surgery or trauma. This study was approved by the Ethics Committee of the Eye, Ear, Nose, and Throat Hospital of Fudan University and in accordance with the tenets of the Declaration of Helsinki. Informed consent was obtained from all of the subjects.

The participants in this study accepted comprehensive ophthalmic examinations, including a biomicroscopic examination, evaluation of Ocular Surface Disease Index Questionnaire (OSDIQ), ST, BUT, and LSCM examinations.

### 2.2. Clinical Investigation

All of the patients were given the OSDIQ to complete, where the 12 items were graded on a scale of 4 to 0 (4, all of the time; 3, most of the time; 2, half of the time; 1, some of the time; and 0, none of the time), and the total OSDIQ score was calculated on a scale of 0 to 100 [18]. After the OSDIQ was completed, a detailed ophthalmological examination was performed in all of the patients. The tear film stability was measured using the BUT with fluorescein and recorded as the mean value of three successive measurements. Additionally, the tear production was determined using the ST without topical anesthesia and expressed as the wet length of the strip for a 5 min measurement. The interval was at least 15 minutes, and all of the examinations were completed in one day by the same investigator.

### 2.3. LSCM Investigation

The Heidelberg Retina Tomograph/Rostock Cornea Module (Heidelberg Engineering GmbH, Dossenheim, Germany) was applied in this study. A 60x water-immersion objective lens and a 670 nm diode laser as a light source were used. The scanning area was 400 mm × 400 mm, with lateral and vertical resolutions of 1 mm each.

Before examination, the eye was topically anesthetized using 0.4% oxybuprocaine hydrochloride (Benoxil; Santen Pharmaceutical, Japan). The patients were asked to position their heads in the headrest and gaze steadily at the fixation tool. The images of the nasal bulbar conjunctivae were taken 5 mm away from the limbus and recorded at one point along the *z*-axis as a single scan. At the end of each examination, one drop of the antibiotic was instilled.

Four parameters were measured using the LSCM. The ECD was studied to investigate the morphology and number of the conjunctival epithelia, and the GCD was studied to investigate the morphology and number of goblet cells. The DCD was studied to investigate the morphology and number of dendritic cells, while the SFD was studied to investigate the morphology and diameter of the subepithelial collagen fibers.

### 2.4. Image Analysis

Three images (without motion blur or compression lines) were selected to calculate the cellular densities of the basal epithelial cells of the conjunctiva (15–25 *μ*m deep), goblet cells (5–25 *μ*m deep), and dendritic cells (5–25 *μ*m deep) using the Cell Count Software (Heidelberg Engineering GmbH) in the manual mode. We selected a square for the region of interest (ROI), and the ROIs of the GCD and DCD were the largest (maximum ROI: 0.1589 mm^2^). The ROI of the ECD was not smaller than one-fourth of the largest ROI. Furthermore, the SFD (25–130 *μ*m deep) analysis method was described in a previous study [16]. The data were expressed as the density ± SD (cells/mm^2^), and all of the images were analyzed by the same investigator. The IVCM operator and the analyzer in this study were two different individuals who were masked with regard to the patients' history and treatment.

### 2.5. Statistical Analysis

The statistical analyses were performed using SPSS 16.0 (SPSS, Chicago, Illinois, USA). The basic descriptive statistics were reported as the means and standard deviations, while the one-way analysis of variance (ANOVA) was used to compare the means of five independent groups, using the post hoc Bonferroni test. A *P* value of less than 0.05 was considered to be statistically significant.

## 3. Results

The demographic profile of the subjects in this study is shown in Table 1, and the mean age and period of treatment were statistically similar between the evaluated groups (*P* > 0.05). There were statistically significant differences found among the control group and the antiglaucoma medication groups in the values of OSDIQ, ST, and BUT (*P* < 0.05), and the clinical data (OSDIQ, ST, and BUT) for the evaluated groups is reported in Table 2. The Bonferroni tests showed that no significant difference was found in the values of the OSDIQ, ST, and BUT between the antiglaucoma groups (*P* > 0.05).

A statistically significant difference was found in the GCD among the five evaluated groups (*F* = 19.464 and *P* = 0.000), and the Bonferroni tests showed significant differences in the GCD between the evaluated groups (*P* < 0.05), with the exception of group 2 and group 3, group 2 and group 5, and group 3 and group 5. This data suggested that the GCD showed a significant reduction in all antiglaucoma therapy groups with respect to the control. Moreover, the prostaglandin group revealed a statistically significant difference when compared with the other glaucoma groups, whereas there was no pronounced difference between the other antiglaucoma therapy groups.

There was a statistically significant difference in the DCD among the five evaluated groups (*F* = 11.295 and *P* = 0.000), and the Bonferroni tests showed that significant differences were found in the DCD between the evaluated groups, except group 2 and group 3, group 2 and group 4, and group 3 and group 4 (*P* < 0.05). This data suggests that the DCD of the subjects in the glaucomatous therapy groups was significantly higher than that in the control. Furthermore, the monotherapy group showed a statistically significant difference from the combination therapy group. However, there was no significant difference revealed between the patients having different monotherapies.

The SFD revealed a statistically significant increase among the five evaluated groups (*F* = 6.721 and *P* = 0.000), and the Bonferroni tests showed that there were significant differences in the SFD between the evaluated groups (*P* < 0.05), with the exception of group 1 and group 4, group 2 and group 3, group 2 and group 5, and group 3 and group 5. This data suggests that the SFD showed a significant increase in all antiglaucoma medication groups with regard to the control group, except for the prostaglandin group. Moreover, a significant decline was found between the prostaglandin group and the other antiglaucoma medication groups, whereas there was no pronounced difference between the other antiglaucoma medication groups.

**Figure 1:**
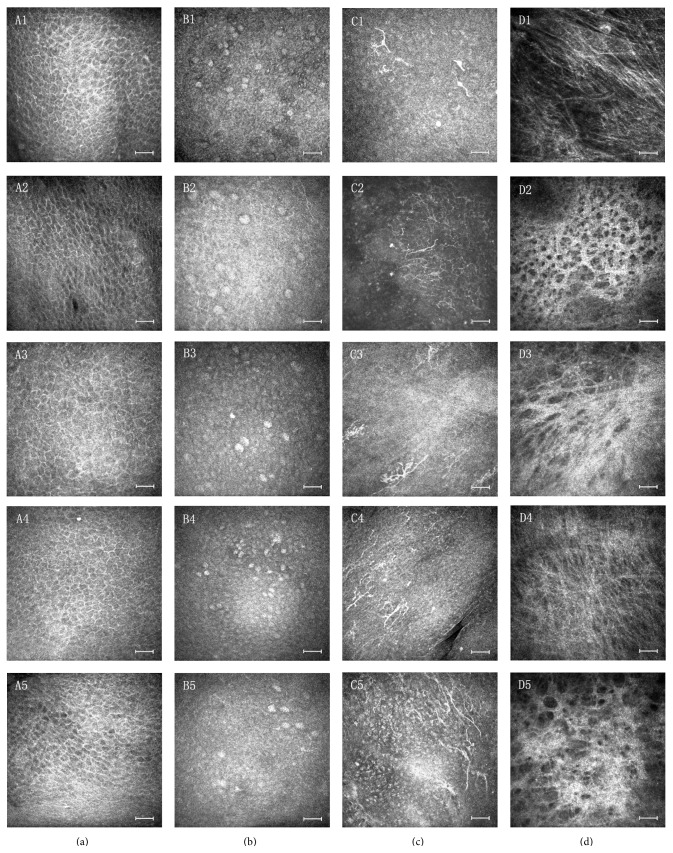
Laser scanning in vivo confocal microscopy of the human conjunctiva in glaucoma patients and control group. (Letter A) Basal epithelial cells of conjunctiva, (Letter B) goblet cells, (Letter C) dendritic cells, and (Letter D) subepithelial fibers. (Number 1) Control group, (Number 2) beta-blockers group, (Number 3) alpha adrenergic agonists group, (Number 4) prostaglandin group, and (Number 5) combination therapy group. The scale bar indicates 50 *μ*m.

No statistically pronounced difference was observed in the ECD between the antiglaucoma medication groups and the control group (*P* > 0.05). The confocal microscopy data (ECD, GCD, DCD, and SFD) for the glaucoma and control groups is reported in Table 3, and the confocal microscopy images are given in Figure 1.

## 4. Discussion

The majority of patients with primary glaucoma must receive medical treatment for most of their lifetimes. Chronic side effects have been drawing more and more attention, among which ocular surface disorders are relatively common. The current study has confirmed the significant changes in the conjunctival structures and tear function with regard to the effects of various antiglaucoma medications. Interestingly, less pronounced changes were found in the prostaglandin group than in the other antiglaucoma medication groups.

As the primary source of ocular mucin, the goblet cells are extremely vulnerable to toxic substances and inflammatory reactions. In the current study, the goblet cells were confirmed to be significantly decreased in all of the antiglaucoma medication groups, with respect to the control. Previously, a similar conclusion was drawn in the histopathology following the long-term effects of antiglaucoma eye drops containing preservatives [8, 21]. Moreover, the mucin expression was found to be reduced due to exposure to the preservative on the human ocular surface [22]. Interestingly, the reduction in the GCD was less pronounced for the prostaglandin group, when compared with the other two monotherapy groups in the current study. Pisella et al. reached a similar conclusion using the impression cytology method [21], while Mastropasqua et al. reported a significant increase in the GCD of glaucomatous patients treated with preservative-free tafluprost, using LSCM and impression cytology [19]. One possible explanation is that the antioxidant properties of the prostaglandins counteract the prooxidative properties of the preservative.

As the strongest antigen presenting cells, dendritic cells express lymphocyte costimulatory molecules and secrete cytokines to initiate immune responses. In the current study, the dendritic cells in the conjunctiva after long-term antiglaucoma therapy were significantly increased, when compared to the control group. These results were in line with those of Sherwood et al., who reported that conjunctival inflammation was documented by the activation and the increase of the dendritic cells [23]. Baudouin et al. reported that the human leucocyte antigen is overexpressed in the dendritic cells of the patients with antiglaucoma medications, when compared with the controls [5, 24].

The DCD of the patients who received multiple therapies was observed to be significantly higher than in those who were on monotherapy in the current study, which was consistent with a previous study in immunohistology [8]. Surprisingly, there was no significant difference revealed in the DCD between the monotherapy groups using LSCM. In one previous study, the preserved prostaglandins were consistently related to less toxic side effects than the BAK counterpart in a conjunctival cell line [25, 26]. However, the dendritic cells served as the strongest antigen presenting cells actively participating in local immunoreactions and might not be useful in evaluating the degree of the inflammatory reaction but may be valuable as an early inflammatory marker in the conjunctiva in vivo.

Subepithelial fibrosis has been demonstrated to develop in patients after the long-term use of antiglaucoma eye drops, likely caused by the increase in the fibroblasts in the subepithelial substantia propria [5, 8]. In the current study, the SFD was measured using LSCM to evaluate the degree of subepithelial fibrosis resulting from different antiglaucoma medications. Interestingly, the mean SFDs in the antiglaucoma medication groups showed a significant increase when compared with the control, with the exception of the prostaglandin group. Similarly, Nuzzi et al. confirmed a significantly high fibroblast density in patients treated with long-term antiglaucoma medications [27]. Furthermore, Terai et al. found that conjunctival specimens receiving latanoprost manifested less inflammatory cell density and stromal collagen density than those receiving timolol, presumably related to the mechanism of the upregulation of MMP-1 and MMP-3 [28]. Prostaglandins might counteract the effects of the preservatives on subepithelial fibrosis and may be beneficial to glaucoma surgery.

As with any observational study, this study had some limitations. The most important was the individual differences. Although we correlated quite a bit of information among the groups, including age, gender, and treatment duration, the patients also differed in the lifestyle and initial condition of the ocular surface, which have great influences on evaluating the effects of antiglaucoma medications. Another limitation of this study was that no preservative-free therapy group was designed, because preservative-free antiglaucoma eye drops were not available in China. This made it difficult to determine the different effects on the conjunctiva between the active compounds and the preservatives of the antiglaucoma medications. Finally, the IVCM method, which was used to determine the GCD, was difficult, with regard to capturing the images and discriminating the goblet cells. Therefore, only one location of the bulbar conjunctiva was selected to observe the GCD, which could pose a significant bias, while the nasal conjunctiva hosted the drugs for a prolonged time period, which might show poor microscopic features. In further studies, several locations should be examined and an average taken in order to minimize the bias.

In conclusion, our study confirmed the significant differences in the conjunctival structures based on the effects of various antiglaucoma therapies. Less pronounced changes were found in the prostaglandin-treated eyes than in the other kinds of antiglaucoma therapies. The GCD, DCD, and SFD might, therefore, become valuable markers to evaluate the side effects of antiglaucoma therapy on the conjunctiva in vivo. Future studies should enroll patients with newly diagnosed glaucoma without any antiglaucoma therapy, design a preservative-free antiglaucoma medication group, and take advantage of both confocal microscopy and impression cytology to evaluate the changes in the conjunctiva.

## Figures and Tables

**Table 1:** Demographic features of control (group 1) and antiglaucoma therapy groups (groups 2, 3, 4, and 5).

	Group 1	Group 2	Group 3	Group 4	Group 5	*P *
*N* eyes	20	20	18	21	21	—
Medication	Control	Beta-blockers	Alpha adrenergic agonists	Prostaglandin	Combination	
Concentration of BAK	—	0.005%	0.005%	0.015%	—	
Gender (male/female)	12/8	12/8	11/7	14/7	13/8	—
Age (yrs)	64.1 ± 15.8	60 ± 13.6	62.6 ± 12.5	61.2 ± 14.0	63.5 ± 15.2	>0.05
Treatment duration (mos)	—	11.6 ± 5.1	11.2 ± 4.5	10.9 ± 4.8	12.6 ± 5.0	>0.05

BAK: benzalkonium chloride; yrs: years; mos: months.

No statistical differences were found between the 5 groups (confidence interval: 95%).

Age and treatment duration data are expressed as mean ± standard deviation and range in parentheses. *P* is by analysis of variance.

**Table 2:** Clinical data comparison between control (group 1) and antiglaucoma therapy groups (groups 2, 3, 4, and 5).

	Group 1 (control)	Group 2 (Bb)	Group 3 (Aa)	Group 4 (Pg)	Group 5 (Ct)	*P *
OSDIQ	8.1 ± 4.7	12.0 ± 10.8	18.6 ± 18.8	17.5 ± 15.6	31.4 ± 16.5	0.000
Schirmer's I test	11.9 ± 4.1	7.7 ± 6.4	9.6 ± 6.2	6.2 ± 3.8	6.2 ± 4.8	0.002
Break-up time	11 ± 2.5	4.7 ± 2.7	5.0 ± 3	3.8 ± 2.4	3.3 ± 1.8	0.000

Bb: beta-blockers group; Aa: alpha adrenergic agonists group; Pg: prostaglandin group; Ct: combination therapy group; OSDIQ: Ocular Surface Disease Index Questionnaire.

Control group showed better clinical results than antiglaucoma therapy groups (confidence interval: 95%).

All data are expressed as mean ± standard deviation. *P* is by analysis of variance.

**Table 3:** In vivo confocal microscopy data between control (group 1) and antiglaucoma therapy groups (groups 2, 3, 4, and 5) (95% confidence interval).

	Group 1 (control)	Group 2 (Bb)	Group 3 (Aa)	Group 4 (Pg)	Group 5 (Ct)	*P *
Epithelial cell density	4299 ± 253	4457 ± 412	4518 ± 380	4547 ± 361	4432 ± 344	0.211
Goblet cell density	408 ± 47	267 ± 61	264 ± 69	336 ± 74	252 ± 77	0.000
Dendritic cell density	15 ± 7	21 ± 8	23 ± 9	22 ± 7	31 ± 8	0.000
Subepithelial fiber diameter	15 ± 4	20 ± 5	20 ± 4	15 ± 4	20 ± 5	0.000

Bb: beta-blockers group; Aa: alpha adrenergic agonists group; Pg: prostaglandin group; Ct: combination therapy group; in vivo confocal microscopy evaluation examined the conjunctival epithelium density, goblet cell density, dendritic cell density (expressed as cells/mm^2^), and subepithelial fiber diameter (expressed as *μ*m).

In vivo confocal microscopy findings demonstrate better results in control than antiglaucoma therapy groups (confidence interval: 95%).

All data are expressed as mean ± standard deviation; *P* is by analysis of variance.
